# An Audit to Determine Whether Managing Non-Cognitive Symptoms of Dementia with Antipsychotics in the Crisis Function Team Is Conducted in Line with the NICE Guidelines

**DOI:** 10.1192/bjo.2026.11704

**Published:** 2026-06-30

**Authors:** Iyad Heja

**Affiliations:** Livewell Southwest, Plymouth, United Kingdom. Hertfordshire Partnership Foundation Trust, Hatfield, United Kingdom

## Abstract

**Aims:**

- To ensure the Southwest Crisis Function Team (in HPFT) manages non-cognitive symptoms of dementia as per NICE guidelines, and provides appropriate non-pharmacological and pharmacological management options.
- If not possible, identify reasons and challenges, and suggest methods for improvement, which could be implemented and re-audited.

**Methods:**

1. All referrals received by CFT in a 12-month period were obtained through the SPIKE database.

2. 48-hour referrals and wrong referrals were excluded.

3. Only service users with a diagnosis of dementia AND were presenting with agitation, aggression, distress or psychosis were included. This information was obtained through PARIS.

4. Service users’ case notes were reviewed to ascertain whether they or their carers:

○ Had a structural assessment and had been offered psychosocial and environmental interventions as initial and ongoing management to reduce distress for service users.

• Had been offered antipsychotics and:

• If they were at the time at risk of harming themselves or others, or were experiencing agitation, hallucinations or delusions that are causing them severe distress.

• If they had discussions about risks and benefits of antipsychotics.

• Had been started on the lowest effective dose possible and were recommended to be reassessed by the prescriber.

**Results:**

1. In 2023, a total of 247 new Service Users (SU) were referred to CFT. After excluding 48-hour follow up referrals and inappropriate referrals, A total of 55 referrals were for SUs who were diagnosed with dementia and were presenting with agitation, aggression, distress, or psychosis. 12 out of the 55 referrals were for 6 SUs who were referred twice at different times during the year.

2. 100% (55/55) of the included referrals received a structural assessment to 1- explore possible reasons for their distress, and 2- check for and address clinical or environmental causes. 100% (55/55) were also offered psychosocial and environmental interventions as initial and ongoing management to reduce distress for SUs.

3. In 12/55 referrals, SUs were already on antipsychotics. In 7/55 referrals, SUs could not be managed without a new prescription of antipsychotics.

4. In these 7 referrals (12.7% of total eligible SUs), SUs received new prescriptions of antipsychotics as a pharmacological intervention and in all 7 referrals, the SUs were at risk of harming self or others**or**were experiencing agitation, hallucinations or delusions that are causing them severe distress.

5. 100% of the 7 referrals showed that SUs were started on the lowest effective dose of the prescribed antipsychotic.

6. However, out of the 7 referrals that received new prescriptions of antipsychotics, none of them (0%) had documentation on PARIS about discussions of risk and benefits of antipsychotics.

7. Finally, when looking at all the referrals where antipsychotics were used (19 referrals in total), there was clear documentation on PARIS about reassessment or a recommendation for reassessment of antipsychotic after 6 weeks in 4/19 referrals (21%).

**Conclusion:**

- 100% of referred SUs received a comprehensive, structural assessment.
- The team offered psychosocial and environmental interventions to all SUs who were eligible.
- The team offered antipsychotics only when necessary, in a small percentage of cases(12.7%), and followed NICE guidelines for initial doses and service user eligibility.
- However, no documentation in any of the service user’s case notes mentioned discussions about risk and benefits of antipsychotics and only 21% had recommendations for reassessment of the antipsychotics within 6 weeks.

